# Psychogenic Nonepileptic Spells in Chronic Epilepsy Patients with Moderate Cognitive Impairment: The Need for Video EEG Monitoring for Adequate Diagnosis

**DOI:** 10.1155/2014/121865

**Published:** 2014-11-19

**Authors:** Diana Mungall Robinson, Batool F. Kirmani

**Affiliations:** ^1^Texas A&M Health Science Center College of Medicine, Bryan, TX, USA; ^2^Department of Neurology, Scott & White Neuroscience Institute, Baylor Scott & White Health, 2401 South 31st Street, Temple, TX 76508, USA

## Abstract

The objective of our study was to emphasize the importance of intensive video EEG monitoring in patients with a well-established diagnosis of epilepsy with moderate cognitive impairment. The idea was to diagnose new onset frequent atypical events prompting the need for frequent emergency room and clinic visits and hospital admissions. Retrospective chart reviews were conducted on patients with chronic epilepsy with moderate cognitive impairment who had an increased incidence of new onset episodes different from the baseline seizures. Data were acquired from electronic medical records. The hospital's Institutional Review Board gave approval for this retrospective analysis of patient records. We retrospectively analyzed data from three patients with an established diagnosis of epilepsy. Extensive chart reviews were performed with emphasis on type and duration of epilepsy and description of baseline seizures and description of new events. There were two men and one woman with moderate cognitive impairment. One subject had generalized epilepsy and other two had temporal lobe epilepsy. The patients were on an average of two to three antiepileptic medicines. The duration of follow-up in our neurology clinic ranged from 9 months to 5 years. The occurrence of increased frequency of these atypical events as described by the caregivers, despite therapeutic anticonvulsant levels, prompted the need for 5-day intensive video EEG monitoring. New atypical spells were documented in all three patients and the brain waves were normal during those episodes. The diagnosis of pseudoseizures was made based on the data acquired during the epilepsy monitoring unit stay. Our data analysis showed that intensive video EEG monitoring is an important tool to evaluate change in frequency and description of seizures even in cognitively impaired patients with an established diagnosis of epilepsy for adequate seizure management.

## 1. Introduction

Epileptic seizures are classified into different types based on the characterization of the events. Careful history is the first step in the diagnosis of epileptic and nonepileptic events. Seizures are due to hyperactivity of the cortical neurons or synchronous neuronal activity in the brain and are further classified into partial and generalized seizures based on electroencephalographic (EEG) data and clinical semiology [1, 2]. However, a diagnostic dilemma arises when patients with established diagnoses of epilepsy develop “Psychogenic Nonepileptic Spells.” Psychogenic Nonepileptic Spells (PNES) are a form of conversion disorder during which patients exhibit seizure like activity but the brain waves are normal. Patients with PNES often have history of abusive past and/or other psychiatric disorders [3]. A careful history is important to detect nonstereotypical spells. If the patients have nonstereotypical events, intensive video EEG monitoring in an epilepsy monitoring unit is the gold standard to capture new spells for definitive diagnosis to rule out pseudoseizures. However, few data are reported in the literature about PNES in this particular subset of patients.

## 2. Rationale

The rationale of our study was to show that chronic epilepsy patients with moderate cognitive impairment can develop pseudoseizures several years after diagnosis of epilepsy, so the need for intensive seizure monitoring should not be disregarded in these patients.

## 3. Methods

Retrospective chart reviews were conducted on three patients with chronic epilepsy with moderate cognitive impairment who had an increased incidence of new onset episodes different from the baseline seizures. Data were acquired from electronic medical records. The hospital's Institutional Review Board gave approval for this retrospective analysis of patient records.

## 4. Results

We report a case series of three patients with an established diagnosis of epilepsy with moderate cognitive impairment.

### 4.1. Case 1

Our first patient was a twenty-eight-year-old Caucasian man with static encephalopathy and generalized epilepsy since the age of five years. He was on multiple medications for control of both staring spells and generalized tonic clonic seizures. He was seen in our epilepsy clinic for increased frequency of staring spells and shaking spells. He does have a history of depression, but no history of abusive past. According to his mother, the spells are different and prolonged as compared to the typical spells. He was admitted to our epilepsy monitoring unit where we were able to document several atypical spells which were nonepileptic in nature. However, his EEG was consistent with generalized epilepsy and one epileptic seizure was detected after sleep deprivation. The study was reviewed with the patient and the caregiver. Follow-up visit was arranged with psychiatry which remarkably reduced the emergency room visits and inpatient hospital admissions.

### 4.2. Case 2

Our second patient was a 45-year-old right-handed Caucasian man with a history of traumatic brain injury in 2008 resulting in moderate cognitive impairment and left temporal partial epilepsy. He also has history of depression since he had traumatic brain injury. He was seen in our epilepsy clinic because of an increased frequency of seizures. The patient was on high doses of three anticonvulsants and continues to remain intractable. According to his mother, he has been having spells on a daily basis and they have to call the ambulance to take him to the hospital. The spells are described by the mother as putting his hands around his head and staring forward for several minutes. During that time, he also becomes unresponsive and this is followed by shaking of the upper extremity. The events may occur and last for about 3 to 5 hours or sometimes most of the day and they have to take him to the emergency room several times a week.

His magnetic resonance imaging demonstrated a significant decrease in signal throughout the left hemisphere, inferring compromised white matter integrity.

The patient was admitted to the epilepsy monitoring unit twice for localization and characterization of seizures secondary to epilepsy and spells of unclear etiology secondary to PNES. We were able to capture the patient's typical spells characterized by unresponsiveness and jerking of the upper extremities not associated with any EEG correlate. The diagnosis of PNES was discussed with the patient and his parents. The patient had an abnormal EEG with potential epileptogenicity in the left temporal region secondary to brain injury; however, the current spells were predominantly PNES. Since his EEG revealed no significant epileptiform abnormalities, the Keppra was discontinued during this admission, and he was sent home on low dose of gabapentin and Topamax for headaches. The study was reviewed with the patient and his parents. Follow-up visits were also arranged with psychiatry which significantly reduced the emergency room visits and inpatient hospital admissions.

### 4.3. Case 3

Our third patient was a 43-year-old right-handed African-American woman with static encephalopathy, manifested by mild ataxia, mental retardation, and complex partial seizures. She has had complex partial seizures with rare secondary generalization since childhood. She also has a history of bipolar disorder but no documented history of physical or sexual abuse. However, during the last few years she started having spells/seizures several times a week or every other week resulting in frequent hospital admissions. She was admitted to our epilepsy monitoring unit twice to characterize her spells for definite diagnosis. We were able to capture spells characterized by tonic stiffening of the whole body with flexion at the elbows, unresponsiveness, followed by jerking. This episode has no EEG correlate, and, therefore, was nonepileptic in nature. Her EEG was consistent with partial epilepsy with potential epileptogenicity in the left temporal region. The study was reviewed with group home staff and she was discharged with a follow-up with psychiatry. The group home staff was educated about PNES which did minimize future emergency room visits.

## 5. Discussion

Epilepsy is a chronic medical condition that poses a huge economic burden and affects the quality of life of both patients and caregivers. The diagnostic dilemma arises when chronic epilepsy patients develop atypical spells that are unresponsive to antiseizure medications. Nine to 15% of all epilepsy patients have concomitant Psychogenic Nonepileptic Spells [3].

The diagnosis requires admission to a specialized unit at an established center referred to as the “epilepsy monitoring unit.” An epilepsy monitoring unit is a hardwired room where patients are monitored continuously by simultaneous video and brainwave “EEG” recording for 4 to 5 days. Diagnosis of Psychogenic Nonepileptic Spells requires demonstration of a negative EEG, meaning absence of “seizure activity” during the entire episode. The other requirement is the careful review of the clinical presentation of the episode since some seizure types, including simple partial seizures, are difficult to capture on a scalp recording.

PNES fall under the somatoform disorders according to the Diagnostic and Statistical Manual of Mental Diseases. Somatoform disorders involve unconscious production of the physical symptoms due to the internal conflicts, which the patient is unable to express. These symptoms are not under the patient's voluntary control and there is no element of intentional faking of the symptoms. Diagnostic and Statistical Manual of Mental Disorders, Fourth Edition (DSM-IV), came up with the new category of conversion disorder for pseudoseizures described as “conversion disorder with seizures.”

PNES have been maintained by the latest volume of the Diagnostic and Statistical Manual of Mental Disorders, Fifth Edition (DSM-V), as a “conversion disorder with attacks or seizures.”

## 6. Conclusion

Our case series suggests that adequate seizure management requires admission to the epilepsy monitoring unit to evaluate change in frequency and description of seizures even in cognitively impaired patients with an established diagnosis of epilepsy.

## References

[1] Fisher R. S., van Emde Boas W., Blume W., Elger C., Genton P., Lee P., Engel J. (2005). Epileptic seizures and epilepsy: definitions proposed by the International League Against Epilepsy (ILAE) and the International Bureau for Epilepsy (IBE). *Epilepsia*.

[2] Dreifuss F. E., Bancaud J., Henriksen O. (1981). Proposal for revised clinical and electroencephalographic classification of epileptic seizures. *Epilepsia*.

[3] Behrouz R., Heriaud L., Benbadis S. R. (2006). Late-onset psychogenic nonepileptic seizures. *Epilepsy & Behavior*.

